# Editorial: Neglected tropical diseases: tackling the challenges of a global world

**DOI:** 10.3389/ftox.2024.1417438

**Published:** 2024-04-30

**Authors:** Armanda Rodrigues, Gabriela Santos-Gomes

**Affiliations:** Global Health and Tropical Medicine, GHTM, Associate Laboratory in Translation and Innovation Towards Global Health, LA-REAL, Instituto de Higiene e Medicina Tropical, IHMT, Universidade Nova de Lisboa, UNL, Lisboa, Portugal

**Keywords:** neglected tropical diseases (NTD), societal impact, integrated control approaches, one health, global health

In today’s interconnected world, the fight against neglected tropical diseases (NTDs) presents both unique challenges and unprecedented opportunities. Despite significant advances in healthcare and medical research, millions of people continue to suffer from NTDs, particularly in low-resource regions, with a disproportionate impact on the most vulnerable populations (Forbes et al., 2023). As we navigate the complexities of a globalized society, it is imperative that we prioritize NTDs as global health problems (WHO, 2022).

Addressing the multifaceted challenges posed by NTDs requires a comprehensive and collaborative approach from all fields of science, and embedded in the One Health concept. From multidisciplinary and integrated scientific research to governments, non-governmental organizations, and the private sector must join forces to develop innovative solutions, strengthen healthcare infrastructures, and improve disease surveillance and control measures.

NTDs perpetuate cycles of poverty, hinder economic growth, and exacerbate existing inequalities (Engels and Zhou, 2020). Moreover, the burden of NTDs extends beyond health systems, impacting education, productivity, and overall quality of life. The first article on this Research Topic (Risat et al.) introduced podoconiosis, a non-infectious geochemical disease prevalent in tropical highlands that causes significant swelling of the lower legs and feet. It affects millions of people worldwide, leading to disability and mental health disorders, exacerbated by the associated social stigma (Deribe et al., 2020). Treatment focuses primarily on managing morbidity and preventing disability, combining biomedical and traditional approaches. However, the social aspects of podoconiosis patients remain largely unexplored. This qualitative study in Huye, Rwanda, investigated the perspectives and experiences of care among patients, family members, traditional healers, and health professionals. Findings highlight the significance of caring networks encompassing various stakeholders, emphasizing not only medical treatments but also the importance of caring relationships. Embedded in care ethics literature, the study highlights the significance of networks and collectives in providing holistic care beyond medical expertise, strongly demonstrating the multidimensionally of NTDs.

Advances in medical research, including the development of novel therapeutics, vaccines, and diagnostic tools, offer promising avenues for combating NTDs. Evidence-based insights that inform public and regulatory frameworks help policymakers to address societal challenges, manage risks, and make informed decisions to promote the wellbeing of communities and contribute to a safe e environment.

In a study examining the first mass drug administration (MDA) campaign for schistosomiasis in a highly urbanized state in Nigeria, Olamiju et al. focused on implementation strategies and highlighted the associated challenges. The implementation of public health MDA campaigns for schistosomiasis is crucial to achieving widespread coverage of preventive treatment, reducing transmission rates, and ultimately controlling the burden of disease in endemic regions (Chanhanga et al., 2023). However, MDA plans must be efficiently designed to achieve their goals. According to the authors, an effective framework for implementing MDA’s plan for hard-to-reach areas should include improved consultation and microplanning, addressing transport Research Topic, community engagement, financial management, stakeholder capacity building, and regular advocacy. These findings provide valuable insights for future schistosomiasis control programs at the sub-district/ward level, in cosmopolitan and urbanized settings like Lagos, Nigeria, and may contribute to the implementation of public policy decisions and monitoring strategies.

Thus, establishing comprehensive strategies for prevention, treatment, and control, as well as ensuring equitable access to healthcare resources and promoting multidisciplinary collaboration among stakeholders are essential in tackling NTDs. Addressing the challenges of another NTD, onchocerciasis, Bhwana et al., evaluated factors associated with low ivermectin uptake. The African Programme for Onchocerciasis Control (APOC), and the Onchocerciasis Elimination Program for the Americas (OEPA), rely on ivermectin to control and eliminate the etiological agent of onchocerciasis (Cupp et al., 2011; Ejere et al., 2012). However, despite two decades of community-directed treatment with ivermectin (CDTI) in Tanzania’s Mahenge district, the prevalence of onchocerciasis remains high among children and adults. This qualitative data was collected in four rural villages identifying several factors associated with persistent transmission. The authors describe reluctance to take ivermectin due to perceived adverse effects and misconceptions, logistical challenges in drug distribution, low awareness of the disease and its association with epilepsy, and inadequate supervision during CDTI. The study recommends optimizing ivermectin uptake through sustained advocacy and improved supervision during CDTI to mitigate onchocerciasis-associated morbidity.

In tackling the challenge of NTDs, the urgency for new therapeutic approaches has fueled the repurposing of approved drugs, which represents a promising strategy for accelerating the development of therapies for NTDs like Chagas’ disease (ChD). ChD remains one of the most significant NTDs, affecting millions of individuals worldwide, and threatening the health and wellbeing of marginalized populations (Bonney, 2014). The emergence of new transmission routes and a deeper understanding of the parasite transmission cycle (Durães-Oliveira et al., 2024) have elevated CD to the forefront of WHO priorities for NTDs. Amiodarone and dronedarone, both antiarrhythmic drugs, have shown potential for treatment of symptomatic cardiac pathology in Chagas’ disease patients. Recent studies have suggested that in addition to their antiarrhythmic effects, these drugs may also have trypanocidal activity against *T. cruzi*, the causative agent of Chagas’ disease (Benaim and Paniz Mondolfi, 2012; Benaim et al., 2020). Francisco et al. conducted a study aimed to evaluate the *in vitro* and *in vivo* activity of these compounds against *T. cruzi* to assess their potential as Chagas’ disease therapies. However, the results indicated that the *in vitro* effectiveness reported in previous studies could not be replicated, and both drugs showed either no activity or cytotoxicity against various mammalian cell lines. *In vivo* efficacy testing in a murine model of *T. cruzi* infection also failed to demonstrate antiparasitic activity at the highest tolerated doses. While the possibility of amiodarone and dronedarone serving as antiarrhythmic agents in Chagas cardiomyopathic patients remains open, their efficacy as trypanocide agents seems unlikely.

Furthermore, as society confronts emerging challenges such as climate change, urbanization, and globalization, the dynamics of NTD transmission and prevalence are evolving. Addressing human migration patterns, exploring alternative treatment strategies, developing prophylactic measures, and recognizing the importance of zoonotic transmission of pathogens are key Research Topic in the fight against neglected tropical diseases, demanding collective action, innovation, and commitment. World Health Organization (WHO) has identified and defined 20 NDTs to be eradicated, eliminated as a public health concern, or controlled by 2030. The 2012 London Declaration on NTDs marks the progress achieved since then and addresses the current challenges in reaching these objectives. Despite the setbacks to NTD programs caused by the COVID-19 pandemic, it has also catalyzed the development of new multisectoral approaches to strengthen health systems. By recognizing the interconnectedness of human, animal, and environmental health, the One Health approach offers a comprehensive framework for addressing NTDs and improving the health and wellbeing of communities worldwide, tackling NTD challenges and building a brighter future for generations to come.
